# Comparison of Cortisol level by Shift Cycle in Korean Firefighters

**DOI:** 10.3390/ijerph17134760

**Published:** 2020-07-02

**Authors:** Ga-Young Lim, Tae-Won Jang, Chang-Sun Sim, Yeon Soon Ahn, Kyoung Sook Jeong

**Affiliations:** 1 Department of Public Health, Hanyang University, Seoul 04763, Korea; limga0076@gmail.com; 2 Department of Occupational and Environmental Medicine, College of Medicine, Hanynag University, Seoul 04763, Korea; om1024@hanmail.net; 3 Department of Occupational and Environmental Medicine, College of Medicine, University of Ulsan, Ulsan 44033, Korea; zzz0202@naver.com; 4 Department of Preventive Medicine, Wonju College of Medicine, Yonsei University, Wonju 26426, Korea; ysahn1203@yonsei.ac.kr; 5 Department of Occupational and Environmental Medicine, Wonju Severance Christian Hospital, Wonju 26426, Korea

**Keywords:** firefighter, shift cycle, shift work, cortisol, circadian rhythm

## Abstract

(1) Study Objectives: By investigating the change of cortisol levels during shift cycles among professional firefighters in Korea, this study aims to evaluate the difference between individuals’ stress response and the recovery of their circadian rhythm after working night shifts. (2) Methods: A total of 325 shift firefighters, who were working in 3, 6, 9, or 21 day cycles, participated in the study. Their urinary and serum cortisol levels were measured during the day (09–18), during the night (18–09), and every 24 h (09–09) per shift cycle, and adjustments were made for confounding factors. (3) Results: Serum cortisol levels were significantly increased after working during the night or for 24 h compared with that of working throughout the day. However, whether working night or 24 h shifts, the serum cortisol levels were undoubtedly different based on the 3, 6, 9, or 21 day cycles. In all shift cycles, the urinary cortisol level decreased during the night or throughout the 24 h shifts compared with sleeping during this time, but this was considered to be significantly applicable only to those working in 21 day cycles. Additionally, in serial measurements, the recovery of urinary cortisol secretion after a night or 24 h shift was successful for individuals working in 9 day cycles, but the recovery was delayed for those working in 6 or 21 day cycles. (4) Conclusions: After analyzing the urine cortisol levels, the study indicates that only subjects working in 9 day cycles fully recovered their circadian rhythm while those working in 6 day or 21 day cycles did not completely recover. Therefore, it is important to recognize how stressful night shifts can be, and it is crucial to enhance firefighters’ current shift cycles in order to allow sufficient recovery of their circadian rhythm as well as the prevention of disrupting their circadian rhythm after working at night. Further research is necessary to take into account the amount of work load, the challenges of being sleep deprived, and the individual’s capacity to overcome sleepiness.

## 1. Introduction

Firefighters serve and protect the community by suppressing fires, rescuing lives, and providing emergency medical service 24/7. In addition, they are exposed to all types of unpredictable and high-tension events due to their job characteristics. Because of their significant role in society, they are recommended to be shift workers, and this, in return, has some negative impact on firefighters. According to Carey et al. [1], 59% of firefighters in US metropolitan cities suffer sleep insufficiency. Also, Mehrdad et al. [2] reported that 69.9% of professional firefighters lack high-quality sleep compared with 37% of the general population of Tehran, the capital of Iran. More specifically, in Korea, Lim et al. [3] addressed that 37% of professional firefighters who are day workers claim they do not sleep well while 51.6% of firefighters who are shift workers state that they do not sleep well. Kim et al. [4] showed that the risk of having poor sleep quality for shift-working professional firefighters is 2.35 times higher than day-working professional firefighters.

Shift work disrupts individuals’ circadian rhythms, as cortisol is one of the major hormones that regulates the circadian rhythm [5,6] and can be affected by the sleep disruption experienced by night shift workers [7]. Cortisol levels change based on the sleep–wake cycle. In general, the cortisol level peaks during the wake time and reaches its lowest point during the first hours of the night sleep episode [8]. Cortisol also plays an important role in mediating and suppressing stress responses [9]. The serum cortisol level is examined to analyze the acute stress response. When individuals are put under stress, their serum cortisol levels are high [10]. The shift work has a great effect on cortisol level changes. In particular, According to Jian [11], the longer the shift workers worked on night shifts, the significantly higher the cortisol level, compared with that of day shift workers. On the other hand, the effect that night shift work has on urinary cortisol is associated with an acute reduction of urinary cortisol production [12]. The constant circadian disruption caused by shift work influences cortisol production, resulting in a flatter pattern over time [13].

According to a prior study, night shift work not only affects the circadian rhythm, but also leads to higher risks of negative health outcomes such as poor sleep quality, short-term memory loss, reduced attention, and increased reaction rate, stress, fatigue, and physiological dysfunction [14,15,16,17]. In the long term, prolonged risks and negative health outcomes can potentially lead to more detrimental results such as cardiovascular disease [18,19,20,21], immune disease [22,23], obesity [24], cancer [25], or other chronic disease. Specifically, for firefighters, insufficient sleep may increase the risk of being injured due to their dangerous, unpredictable work [26]. This can also prevent firefighters from making the proper and effective decisions required to respond to the emergency [15].

Although it is extremely difficult for firefighters to completely avoid shift work given their job characteristics, it is necessary to minimize the disruption of their circadian rhythm by modifying the shift cycle [15]. An appropriate shift cycle can be designed to allow firefighters to restore their normal sleep patterns and take sufficient rest between their duties [27,28,29]. According to Billings and Focht [15], it takes at least two days to recover from inevitable shift works to return to the normal circadian rhythm, but it takes at least four days if there has been a severe alteration to the circadian rhythm.

In Korea, most professional firefighters at the same fire station work the same shift cycle, while among the various fire stations there are the 3 day, 6 day, 9 day, and 21 day shift cycles. It is necessary to determine which shift cycle is healthier for the individuals and more appropriate for their circadian rhythm. In order to identify the optimal shift cycle for the professional firefighters in Korea, we evaluated their stress response and the recovery status of their circadian rhythm after working night shifts by analyzing the change of their cortisol level per shift cycle.

## 2. Materials and Methods

### 2.1. Participants

**Figure 1:**
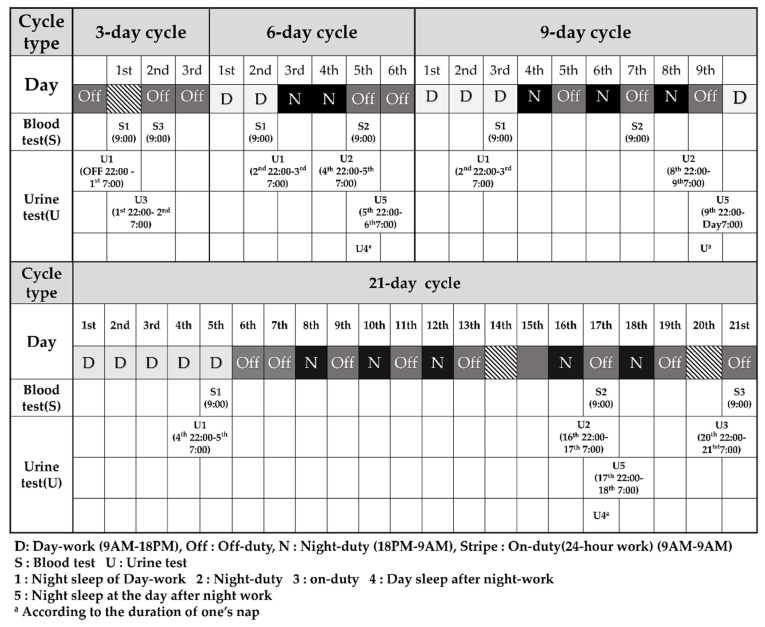
The work schedule by shift cycle.

**Figure A1:**
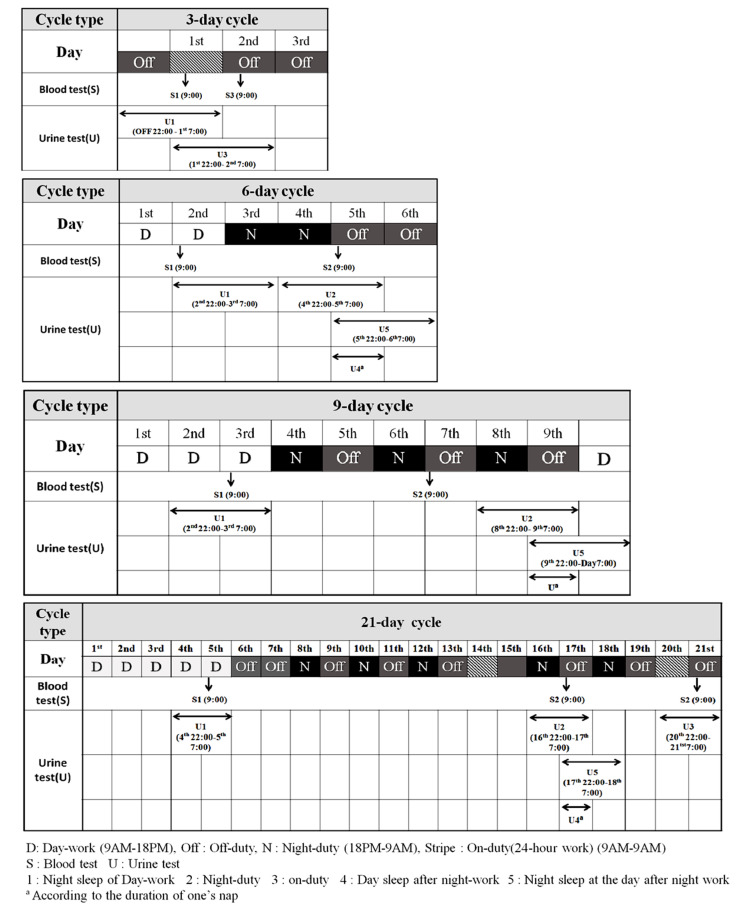
The work schedule by shift cycle.

This research is a part of the SLEep Panel Study (SLEPS), which is designed to improve sleep problems and determine the appropriate shift cycle for Korean firefighters. In SLEPS, study subjects were randomly selected from a total of eight Korean fire stations, from which one or two fire stations were from each of the following general regions: urban, suburban, and rural areas. A total of 516 firefighters were enrolled in SLEPS; 88 firefighters were day workers while 428 of them were shift workers. Shift work consisted in 3, 6, 9, and 21 day cycles. The work schedule in accordance with the shift cycle is shown in Figure 1. Detailed pictures can be found in Figure A1 (Appendix B). All firefighters from the same fire station have the exact same shift cycle, but the fire station consists of three shift teams. Each shift team follows the same schedule. For example, there are A, B, and C teams in 3 day cycle, and A, B, and C teams work at first, second, and third day, respectively. A team works at first day and then has off duty for 2 days.

Respectively, the number of participants by shift cycle was 75, 88, 93, and 172 firefighters by 3, 6, 9, and 21 day cycle. In this study, we measured the serum and urinary cortisol level of 325 firefighters who were shift workers and included their response to the provided questionnaires. We excluded any subject missing general demographic or occupational variables. After taking all these factors into consideration, the respective number of participants by shift cycle was 58, 57, 68, and 142 firefighters by 3, 6, 9, and 21 day cycle.

### 2.2. Procedures

Two professional nurses administered the questionnaires and addressed the study protocol to the firefighters at the time of enrollment. Figure 1 illustrates the study protocol. The firefighters completed the questionnaires with a pen on a given sheet of paper on the first day of participation. From the questionnaires, we collected general data of the participants, such as their occupational and health characteristics. Also, we drew their blood and collected their urine for the analyses of their cortisol levels. Tests were performed according to the schedule for the shift cycle.

This study protocol was approved by the Institutional Review Board Committee of Dongguk University Ilsan Hospital (DUIH 2017-08-014-001) and Yonsei University Wonju Severance Christian Hospital (IRB No. CR318031). Before starting our research, we explained the procedure and the study objective to the subjects and received a written informed consent from all of the participants.

### 2.3. Measures

#### 2.3.1. General Demographic and Occupational Variables

We surveyed the subjects’ age, gender, education, marital status, and income. Occupational characteristics were surveyed to have knowledge of their specific role (fire suppression, emergency medical service, rescue, fire investigation) and to determine their shift work schedule (3, 6, 9, and 21 day cycle). We recognized that firefighters who work 24 h shifts work from 9 AM to 9 AM the following morning. Lastly, firefighters who work during the night work from 9 AM to 6 PM. During night work, firefighters work from 6 PM to 9 AM the following morning.

#### 2.3.2. Health-Related Variables

We surveyed the subjects’ alcohol consumption, smoking habit, caffeine intake, and subjective health conditions for confounding variables that can potentially impact their sleep or health status. Alcohol consumption was categorized as “non-drinker” or “drinker”. Smoking habit was categorized as “non-smoker”, “past-smoker”, or “smoker”. The caffeine intake variable was categorized as “Yes” or “No”. The subjective health conditions variable was categorized as “very good”, “good”, “normal”, “bad”, or “very bad”. To consider the individual’s psychological health factors that could affect their sleeping pattern and sleep quality, we assessed depression, anxiety, fatigue, and chronotype with the Patient Health Questionnaire-9 (PHQ-9) [30,31], Generalized Anxiety Disorder-7 (GAD-7) [32,33], Fatigue Severity Scale (FSS) [34,35], and the Korean-translated Composite Scale (KtCS) [36,37]. To take into consideration any work-related psychological disorder these firefighters may have developed, we used the Primary Care Post-Traumatic Stress Disorder Screen (PC-PTSD) [38] to survey whether they developed post-traumatic stress disorder (PTSD) [39] or not. The subjects were categorized as having PTSD if they answered yes to three or more out of the four questions provided during PC-PTSD [38]. To diagnose depression amongst the subjects, PHQ-9 was developed by Kroenke et al. [30] and translated into Korean by Park et al. [31]. This questionnaire is composed of nine criteria, which are each scored from zero to three points. An individual is diagnosed with depression if their score is greater than or equal to five points. Furthermore, anxiety disorder was diagnosed with a score greater than or equal to five points from GAD-7 [33], in which each criteria is also scored from zero to three points. The Fatigue Severity Scale (FSS) by Krupp et al. [34] consists of nine criteria as well and is scored from one to seven points. Fatigue was diagnosed with a score greater than or equal to 10 points [34,35]. Chronotype, also known as morningness–eveningness, was measured by a Korean version of the composite scale that was originally developed by Smith et al. [36], KtCS [36,37]. It consists of thirteen criteria, where each one is scored from one to four or five points. The chronotype of an individual is classified as eveningness if the score is less than or equal to twenty-seven, intermediate if the score ranges between twenty-eight and forty, and morningness if the score is greater than or equal to forty-one [40].

To assess the subjects’ sleep patterns and quality, we evaluated their Insomnia Severity Index (ISI) [41], Epworth Sleepiness Scale (ESS) [42], and Pittsburgh Sleep Quality Index (PSQI) [43]. ISI, developed by Morin et al. [41], evaluates the subjects with seven criteria, where each one is scored from zero to four points and individuals with a score greater than or equal to eight points are considered to have insomnia. Daytime sleepiness was determined by ESS, which contains eight criteria, and each one is scored from zero to three points. Individuals with a score greater than or equal to ten points were diagnosed with daytime sleepiness [42]. Lastly, PSQI, developed by Buysse [43], consists of nineteen questions which are self-rated to evaluate individuals’ subjective opinion on their sleep quality over the previous month before the study. The 19 questions are divided into seven categories: subjective quality of sleep, sleep incubation, duration of sleep, habitual sleep effects, sleep disturbance, use of sleeping pills, and daytime functional disorders, with a PSQI score less than or equal to five points being considered normal sleep quality and a PSQI score greater than five points being assessed as poor quality of sleep [43,44].

#### 2.3.3. Hormone Measurement

Figure 1 showed when we sampled the blood and collected the urine to investigate the hormonal changes based on the working schedule of each shift cycle. The serum cortisol level was measured at 9 AM regardless of whether the individual worked or slept that previous night, to investigate the stress response for night work and compare it to the baseline. The urinary cortisol level was measured from 10 PM to 7 AM in order to collect the first urine of the day to compare the difference between the individuals who work at night or for 24 h and those who sleep at night to work during the day.

Blood samples were obtained by venipuncture of the antecubital vein with the individual in a sitting position to eliminate possible positional effects [45]. The blood was sampled at 9 AM to consider diurnal variation and feasibility. For individuals working day shifts, their blood sample was drawn at 9 AM on the last day shift for each shift cycle. The serum cortisol level measured by this method is classified as sC1. After the second night shift in the 6 and 9 day cycles and the first night shift of the second week in the 21 day cycle, the blood samples were collected at 9 AM. The serum cortisol level measured during these times is classified as sC2. The blood sample was drawn at the last 9 AM of the 3 and 21 day cycles for individuals working 24 h shifts, and this measurement of the serum cortisol level is classified as sC3.

In order to collect the subjects’ urine, urine packs were distributed and participants were informed to collect their urine at a given time. All subjects were asked to void bladder right before their first urine collection of the day. The subjects collected their urine according to the specific time intervals they were told to follow. For individuals working day shifts in 3 day cycles, urine was collected from 10 PM on the second day off to 7 AM the following day. For those working in 6, and 9 day cycles, urine was collected from 10 PM on the second day of work to 7 AM the following day. For those working in 21 day cycles, the urine was collected from 10 PM on the fourth day of work to 7 AM the following morning. The urine cortisol levels collected from day workers were labeled as uC1. For individuals working during the night in 6, and 9 day cycles, the urine cortisol levels were measured with urine collected from 10 PM during their last night shift of the cycle to 7 AM the following morning. For those working at night in 21 day cycles, their urine was collected on the first night of the second week of their night shift. These urine cortisol levels were labeled as uC2.

In addition, for individuals working 24 h shifts in 3 and 21 day cycles, the urine was collected from 10 PM on the day of the 24 h shift to 7 AM the following morning. These urine cortisol levels were labeled as uC3.

Lastly, in order to measure the hormonal change during the recovery process of individuals’ circadian rhythm after their night duty, their urine was sampled right after their night duty, and these were labeled as uC4. More urine was collected the next day from 10 PM to 7 AM the following morning, and these were labeled as uC5.

The serum cortisol level was analyzed by ADVIA Centaur XP (Siemens, Washington, DC, USA). Analysis of the collected urinary cortisol levels was implemented by Cobas 8000 e602 (Roche Diagnostics, Rotkreuz, Swiss). The analyses were completed in the Seoul clinical laboratories which are certified locations of assessment by the European quality assurance for research and development for medical exam and diagnosis.

### 2.4. Statistical Analyses

Chi-square tests were performed to check the distribution of general characteristics by shift cycles, which are categorical variables. An analysis of variance (ANOVA) was performed for continuous variables such as age. The Kolmogorov–Smirnov test was conducted to verify the normal distribution of the cortisol. The distributions of the serum and urinary cortisol levels were skewed to the right, so we transformed the data into a logarithm with base 2. The log-transformed data was then normally distributed. For the log-transformed data, a paired t-test was performed to compare the serum and urinary cortisol levels for day and night work. The linear mixed model (LMM) was used to then confirm the difference in the repeated measurements of the urinary cortisol levels according to each shift cycle. In addition, analysis of covariance (ANCOVA) was conducted to compare the cortisol levels by shift cycles and to identify confounding factors that affect cortisol changes at each measurement point per shift cycle. As a result, gender, age, chronotype, depression, job, PTSD, sleep disorder, fatigue, caffeine intake, subjective health condition, and sleep quality were confounding factors that we adjusted through LMM. The SAS9.1 program (SAS Institute Inc., Cary, NC, USA) was used for statistical analyses.

## 3. Results

### 3.1. Distribution of General Demographic Parameters and Health-Related Habits

General characteristics by shift cycles are shown in Table 1. There were significant differences in each individual’s age (*p* = 0.003), education level (*p* = 0.001), marital status (*p* = 0.041), income (*p* = 0.001), and job (*p* < 0.001), whereas gender, alcohol drinking, caffeine intake, and smoking status were not as significantly different by shift cycles. After comparing the means of age between each cycle, the results of the post-analysis are shown in Table A1 (Appendix A).

Distribution of psychological health by shift cycle is shown in Table 2. Of all the variables, only the sleep quality (PSQI) variable showed significant difference by the shift cycle (*p* < 0.001).

### 3.2. Changes of Hormone at Each Shift Cycle

The difference of serum cortisol level by working time and shift cycles is shown in Table 3. In all shift cycles, the serum cortisol level significantly increased after night or 24 h shifts compared with the level measured after day shift (*p* < 0.01). More specifically, the serum cortisol levels were significantly higher in individuals working in 6 and 9 day cycles than in those working 3 and 21 day shift cycles when measured after their night or 24 h shifts (*p* = 0.002). However, there were no significant differences in serum cortisol levels measured after working during the day and sleeping at night (*p* = 0.633) in all shift cycles. Even after adjustments of confounding factors, the serum cortisol levels were significantly different after night or 24 h work by shift cycle (*p* = 0.007).

The difference of urinary cortisol level by working time and shift cycles is shown in Table 4. There were no significant differences in urinary cortisol level at any working time by shift cycle. For individuals working in 3, 6, and 9 day cycles, there were no significant differences in their urinary cortisol levels between sleeping at night after day work and working night or for 24 h (*p* = 0.884, 0.280, and 0.173 in each shift cycle, respectively). However, for subjects working at night in 21 day cycles, their urinary cortisol levels significantly decreased compared with those sleeping at night after working during the day (*p* = 0.001). For individuals working in 21 day cycles, their urinary cortisol levels also decreased when measured during their daytime sleep after their night work compared with the measurements during their night sleep after their day work, but this difference was not statistically significant (*p* = 0.112). In addition, for individuals working in 6 and 21 day cycles, their urinary cortisol levels measured during their night sleep after their night shift the day before significantly decreased compared with subjects’ urinary cortisol levels measured during their night sleep after their day shift (*p* = 0.050, 0.011, and 0.007, respectively).

The urinary cortisol levels by working time (sleeping at night after day work, night or 24 h work, night sleep in the next day after night work) in 6, 9, and 21 day cycle are shown in Table 5. The urinary cortisol levels decreased during night or 24 h shift compared with that of those sleeping during that time and working during the day. However, the urinary cortisol level fully recovered back to its original state for those working in 9 day cycles, but still remained lower for those working in 6 (although not significant, *p* = 0.172) and 21 day cycles (*p* = 0.002).

## 4. Discussion

This study was designed to develop a shift schedule that is physiologically appropriate and accommodating, by investigating the changes of cortisol levels of Korean firefighters by shift cycles. We compared the difference between individuals’ hormone levels by shift cycles. Specifically, we collected and analyzed the serum and urinary cortisol levels of firefighters by shift schedules.

The urinary cortisol levels were different by shift schedule, and we concluded that the recovery of the urinary cortisol level for individuals working in 21 day cycles was delayed compared with those working in 9 day cycle.

In all shift cycles, there was no difference in the serum cortisol levels measured after working during the day and sleeping at night, but the serum cortisol level was significantly different by shift cycles when measured after working at night or for 24 h. In addition, the serum cortisol level was significantly higher after working at night or for 24 h than after sleeping at night in all cycles (*p* ≤ 0.001). These results suggest that these shift-working firefighters are under more stress than those who work day shifts and sleep at night. These results also indicate that stress levels vary depending on the shift cycle. Many previous studies indicate that the cortisol response is positively related to work stress [46,47] and the increase of serum cortisol levels indicates acute stress [10]. The Dutch study found that in a span of four to fourteen months after a group of police officers commenced shift work, their morning salivary cortisol levels significantly rose [48]. Li et al. also found that waking cortisol levels was significantly higher among physicians who are shift workers than among those who are not [11].

Urinary cortisol levels did not differ by shift cycles when measured during individuals’ night sleep after day work and during night or for 24 h shifts. However, the level significantly decreased during night work in the 21 day cycle and remained significantly lower during night sleep of the next day. Thus, in the 21 day cycle, the urinary cortisol level was incompletely recovered and this strongly influenced the sleep hormones. The term incomplete recovery is used to describe significantly higher or lower concentrations of certain categories compared with its baseline [49]. Similar to our findings, the Canadian study found that less urinary cortisol was produced during the night shift cycle when comparing the diurnal cortisol secretion of shift workers by working time (day work or night work) [12]. The attenuation of cortisol production in shift workers may attribute to the decreased urinary cortisol level during the night shift cycle.

We observed the serial changes of urinary cortisol levels caused by shift work. The urinary cortisol level of individuals working in 9 day shift cycles had fully recovered, while the levels of those working in 6 and 21 day shift cycles had not returned to its original state. In particular, there was a significant difference identified in the 21 day cycle. As a result, we found that the 21 day cycle demonstrates the greatest influence and slowest recovery from the hormonal change.

Many previous studies suggest that night shift work often leads to lack of sleep, circadian disruption, and contributes to fatigue and increased strain on the body [50,51]. Therefore, on a personal precautionary note, firefighters should make efforts to get quality sleep at home while being off duty in order for their biorhythms to recover smoothly [52]. In addition, appropriate intervention is needed to organize a shift schedule where shift workers can continue working without their health being impaired [53].

In cases of frequent interruption of the normal circulatory rhythms, the body requires at least 2 days and can take up to 3–4 days for severely disturbed circadian rhythmicity from shift work to fully recover [54,55]. In a study by Billings [15], in the 24 on-48 off shift schedule where two off days are provided between one shift, they believe that this schedule allows the circadian rhythm to return to normal. Research done by Frazier [56] recommended the 3 day cycle (24 on-48 off) as a long-term solution covering efficiency, productivity, and health and safety concerns. In our study as well, there was the least amount of hormonal change in the 3 day cycles, while there was a much greater amount of change in the 21 day cycles compared with the other shift cycles.

There are several limitations in this study. (1) The different age distribution by shift cycle, which may influence individuals’ education level, marital status, and income distribution. (2) Not all urinary cortisol levels were tested each time because not all subjects collected urine on every shift schedule. Lastly, (3) we were not able to measure the amount of job demands during night or 24 h shifts.

However, our research has originality as we took initiative to compare the difference in the serum cortisol level and the serial changes of urinary cortisol level by shift schedule within shift workers, by measuring each participant’s sleep hormones between their night sleep after day work and night work. Overall, our elaborate method and procedures contribute to a more reliable and outstanding result compared with a conventional survey method as we randomly selected the subjects from fire departments nationwide and analyzed the subjects’ hormone level to determine their quality of sleep.

## 5. Conclusions

From the research, it is understood that night work can be stressful to many individuals, especially firefighters who serve such an important role in the community. In terms of circadian rhythm recovery through urine cortisol levels, circadian rhythms were fully recovered in the 9 day cycle, but were not completely recovered in the 6 day and 21 day cycles. Therefore, it is necessary to improve the shift cycles of firefighters to allow sufficient recovery from working throughout the night. Further study is needed, taking into account the amount of work load, the challenges of sleep deprivation, and the individual’s capacity to overcome sleepiness.

## Figures and Tables

**Table 1:** Distribution of general demographic parameters by shift cycle (*N* = 325).

Variables	3 Cycle		6 Cycle		9 Cycle		21 Cycle		*p*
	*N*	(%)	*N*	(%)	*N*	(%)	*N*	(%)	
Age (years)									0.003
20–29	1	1.7	1	1.8	6	8.8	21	14.8	
30–39	18	31.0	26	45.6	24	35.3	58	40.9	
40–49	29	50.0	19	33.3	22	32.4	50	35.2	
50–59	10	17.2	11	19.3	16	23.5	13	9.2	
Mean ± SD	42.8 ± 7.07		41.4 ± 8.54		41.9 ± 8.56		38.4 ± 8.12		0.001
Gender									0.299
Male	51	87.9	55	96.5	64	94.1	133	93.7	
Female	7	12.1	2	3.5	4	5.9	9	6.3	
Education									0.001
High school	6	10.3	19	33.3	12	17.7	22	15.5	
College (2 years)	22	37.9	18	31.6	11	16.2	45	31.7	
University (4 years)	30	51.7	20	35.1	45	66.2	75	52.8	
Marital status									0.041
Married	49	84.5	47	82.5	53	77.9	97	68.3	
Single	9	15.5	10	17.5	15	22.2	45	31.7	
Income (1000 Korean won/month)									0.001
<3000	9	15.5	15	26.3	9	13.2	45	31.7	
3000–5000	25	43.1	30	52.6	38	55.9	73	51.4	
≥5000	24	41.4	12	21.1	21	30.9	24	16.9	
Job									<0.001
Fire suppression	19	32.8	23	40.4	35	51.5	83	58.5	
EMS *	11	19.0	11	19.3	22	32.4	34	23.9	
Rescue	16	27.6	14	24.6	7	10.3	10	7.0	
Fire investigation	12	20.7	9	15.8	4	5.9	15	10.6	
Alcohol drinking									0.675
Non-drinker	12	20.7	9	15.8	15	22.1	23	16.2	
Drinker	46	79.3	48	84.2	53	77.9	119	83.8	
Smoke									0.514
Non-smoker	20	34.5	22	38.6	25	36.8	43	30.3	
Past-smoker	22	37.9	23	40.4	19	27.9	55	38.7	
Smoker	16	27.6	12	21.1	24	35.3	44	31.0	
Caffeine Intake									0.268
No	7	12.1	12	21.1	13	19.1	17	12.0	
Yes	51	87.9	45	79.0	55	80.9	125	88.0	
Subjective health condition									0.869
very good	4	6.9	1	1.7	4	5.8	6	4.2	
good	22	37.9	25	43.9	28	41.2	59	41.6	
normal	28	48.3	25	43.9	32	47.1	70	49.3	
bad	4	6.9	6	10.5	4	5.9	6	4.2	
very bad	0	0.0	0	0.0	0	0.0	1	0.7	

* EMS: Emergency medical service.

**Table 2:** Distribution of psychological health by shift cycle.

Variables	3 Cycle		6 Cycle		9 Cycle		21 Cycle		*p*
	*N*	(%)	*N*	(%)	*N*	(%)	*N*	(%)	
PTSD									0.662
No	52	89.7	54	94.7	63	92.7	134	94.4	
Yes	6	10.3	3	5.3	5	7.3	8	5.6	
KtCS									0.694
Evening type	2	3.4	1	1.8	4	5.9	9	6.3	
Intermediate type	44	75.9	41	73.2	53	77.9	107	75.4	
Morning type	12	20.7	14	25.0	11	16.2	26	18.3	
Depression									0.138
No	56	96.5	54	94.7	68	100	136	95.8	
Yes	2	3.5	3	5.3	0	0.0	6	4.2	
Insomnia									0.988
No	52	89.7	51	89.5	62	91.2	128	90.1	
Yes	6	10.3	6	10.5	6	8.8	14	9.9	
Daytime sleepiness									0.571
No	53	91.4	53	93.0	63	92.6	130	91.5	
Yes	5	8.6	3	5.3	4	5.9	12	8.5	
No answer	0	0.0	1	1.7	1	1.5	0	0.0	
Fatigue									0.944
No	33	56.9	31	55.4	41	60.3	79	56.4	
Yes	25	43.1	25	44.6	27	39.7	61	43.6	
Anxiety									0.245
No	56	96.5	54	94.7	67	98.5	140	98.6	
Yes	2	3.5	1	1.8	1	1.5	2	1.4	
No answer	0	0.0	2	3.5	0	0.0	0	0.0	
Sleep quality									<0.001
No	12	20.7	8	14.0	20	29.4	49	34.5	
Yes	30	51.7	23	40.4	36	52.9	79	55.6	
No answer	16	27.6	26	45.6	12	17.7	14	9.9	

PTSD was measured with PC-PTSD (Primary Care Post-Traumatic Stress Disorder): answered yes at ≥ 3 questions; KtCS (Korean-translated Composite Scale): eveningness ≤ 27, intermediate 28–40, and morningness ≥ 41; depression was measured with PHQ-9 (Patient Health Questionnaire-9): Yes (≥5 points); insomnia was measured with ISI (Insomnia Severity Index): Yes (≥8 points); daytime sleepiness was measured with ESS (Epworth sleepiness scale): Yes (≥10 points); fatigue was measured with FSS (Fatigue Severity Scale): Yes (≥10 points); anxiety was measured with GAD-7 (Generalized Anxiety Disorder-7): Yes (≥5 points), No (<5 points); sleep quality was measured with PSQI (Pittsburgh Sleep Quality Index): Yes (>5 points), No (≤5 points).

**Table 3:** The difference of serum cortisol level by working time and shift cycles (unit: μg/dL).

Shift Cycle	*N*	sC1	sC2/sC3	*p*
		GM ± GSD	GM ± GSD	
3	58	2.42 ± 0.51	2.69 ± 0.33	0.001
6	57	2.47 ± 0.38	2.77 ± 0.26	<0.001
9	68	2.48 ± 0.30	2.81 ± 0.23	<0.001
21	142	2.42 ± 0.33	2.64 ± 0.34	<0.001
Total	325	2.44 ± 0.37	2.71 ± 0.31	<0.001
*p*		0.633	0.002	
*p* ^†^		0.714	0.007	

^†^*p*-value after adjustment for gender, age, chronotype, depression, job, PTSD, sleep disorder, fatigue, caffeine intake, subjective health condition, sleep quality. sC1: serum cortisol after night sleep after day work. sC2: serum cortisol after night work. sC3: serum cortisol after 24 h work.

**Table 4:** The difference of urinary cortisol level by working time and shift cycles (unit: μg/dL).

Shift Cycle	*N*	uC1	uC2/uC3	uC4	uC5	*p*
		GM ± GSD	GM ± GSD	GM ± GSD	GM ± GSD	
3	57	1.33 ± 0.90	1.31 ± 1.75			0.884
6	51	1.34 ± 0.84	1.14 ± 1.02			0.280
9	60	1.39 ± 0.80	1.16 ± 0.97			0.173
21	123	1.38 ± 0.96	0.98 ± 0.85			0.001
Total	291	1.37 ± 0.89	1.12 ± 0.89			0.001
	*p*	0.985	0.133			
	*p* ^†^	0.867	0.093			
21	61	1.34 ± 0.85		1.06 ± 1.02		0.112
6	42	1.42 ± 0.79			0.95 ± 1.31	0.050
9	54	1.38 ± 0.83			1.43 ± 1.05	0.766
21	121	1.37 ± 0.95			1.04 ± 1.04	0.011
Total	217	1.39 ± 0.89			1.13 ± 1.11	0.007
	*p*	0.963			0.052	
	*p* ^†^	0.799			0.051	

^†^*p*-value after adjustment for gender, age, chronotype, depression, job, PSTD, sleep disorder, fatigue, caffeine intake, subjective health condition, sleep quality. uC1: urinary cortisol during night sleep after day work. uC2: urinary cortisol during night work. uC3: urinary cortisol during 24 h work. uC4: urinary cortisol during the day sleep right after the night duty. uC5: urinary cortisol during night sleep in the next day after night work.

**Table 5:** The difference of urinary cortisol level between shift cycles of shift-working firefighters (unit: μg/dL).

Shift Cycle	*N*	uC1	uC2/uC3	uC5	*p*	*p* ^†^
		GM ± GSD	GM ± GSD	GM ± GSD		
6	42	1.42 ± 0.79	1.18 ± 0.99	0.95 ± 1.31	0.128	0.172
9	54	1.38 ± 0.83	1.08 ± 0.97	1.43 ± 1.05	0.117	0.105
21	121	1.37 ± 0.95	0.98 ± 0.86	1.04 ± 1.04	0.003	0.002
Total	217	1.39 ± 0.89	1.05 ± 0.91	1.13 ± 1.11	0.001	0.001
*p*		0.963	0.443	0.052		
*p* ^†^		0.799	0.300	0.051		

^†^*p*-value after adjustment for gender, age, chronotype, depression, job, PSTD, sleep disorder, fatigue, caffeine intake, subjective health condition sleep quality. uC1: urinary cortisol during night sleep after day work. uC2: urinary cortisol during night work. uC3: urinary cortisol during 24 h work. uC5: urinary cortisol during night sleep in the next day after night work.

## Appendix A

**Table A1:** Mean age by shift cycle with post hoc test Scheffe.

Variables	3 Cycle	6 Cycle	9 Cycle	21 Cycle	F	*p*	Scheffe
	Mean ± SD	Mean ± SD	Mean ± SD	Mean ± SD			
Age (years)	42.8 ± 7.07	41.4 ± 8.54	41.9 ± 8.56	38.4 ± 8.12	5.77	0.001	1.3 > 4

1 = 3 cycle, 2 = 6 cycle, 3 = 9 cycle, 4 = 21 cycle.
